# Interaction between Negatively Charged Fish Gelatin and Cyclodextrin in Aqueous Solution: Characteristics and Formation Mechanism

**DOI:** 10.3390/gels7040260

**Published:** 2021-12-13

**Authors:** Qi Fang, Nao Ma, Keying Ding, Shengnan Zhan, Qiaoming Lou, Tao Huang

**Affiliations:** College of Food and Pharmaceutical Sciences, Ningbo University, Ningbo 315800, China; 18268537580@163.com (Q.F.); 15891285779@163.com (N.M.); kk0103102021@163.com (K.D.); louqiaoming@nbu.edu.cn (Q.L.)

**Keywords:** fish gelatin, cyclodextrin, stable soluble complex, gel strength, secondary structure

## Abstract

The effect that ratios of fish gelatin (FG) to α/β/γ cyclodextrins (α, β, γCDs) had on the phase behavior of a concentrated biopolymer mixture were comparatively investigated. This showed that the formed biopolymer mixture had the highest gel strength at ratios of FG–CD = 90:10. FG could interact with CDs to form stable soluble complexes with lower values of turbidity, particle size and ζ-potential. All of the FG–CD mixture solutions exhibited pseudo-plastic behaviors, and FG–αCD samples had the highest viscosity values than others. The addition of CDs could unfold FG molecules and make conformation transitions of FG from a random coil to β-turn, leading to the environmental change of hydrophobic residues and presenting higher fluorescence intensity, especially for βCDs. FTIR results revealed that the formation of intermolecular hydrogen bonds between FG and CD could change the secondary structure of FG. These findings might help further apply FG–CD complexes in designing new food matrixes.

## 1. Introduction

Proteins and polysaccharides have been widely used as functional ingredients as natural biopolymers in the food industry [1]. The interactions between the two macromolecular substances make protein-polysaccharide mixtures have better functional properties (e.g., emulsifying, emulsifying stability, foaming, water retention, thermal stability and oxidation resistance) than those of pure protein or polysaccharide systems [2]. Therefore, protein-polysaccharide mixtures have broad applications in the food industry as surfactants, for textural modification and fat replacement [3]. Generally speaking, the interactions between proteins and polysaccharides can be associative or segregative [4]. When proteins and polysaccharides have opposite charges, associative electrostatic interactions make proteins and polysaccharides easily form complexes through electrostatic attraction [3,5]. It was also found that when both biopolymers were net-negatively charged, complexes could still form [3]. The formed biopolymers still have complex coacervation behaviors, which are mainly determined by the electrostatic interactions, such as molecular properties (charge density and size), environmental factors (pH, mixing ratio of proteins and polysaccharides, salt, total concentration of biological macromolecules and temperature) and other factors (shear rate) [5,6].

Fish gelatin (FG) is a kind of gelatin extracted from fish skins or bones. As a potential substitute to mammalian gelatin, FG has attracted extensive interest over the years because there is no risk of bovine spongiform encephalopathy or foot-and-mouth disease, and it meets the requirements of Kosher and Halal dietary regulations [7]. Fish gelatin-polysaccharide complexes have been widely investigated in food science, mainly on their gelation, rheology, interfacial activity and stability. Sow et al. [8] reported that fish gelatin and low concentrations of sodium alginate could form stable complex coacervates through the electrostatic interactions, improving gel strength and the hardness of gelatin gels. Huang et al. [9] reported that the viscosity of fish gelatin-anion polysaccharide composite solutions increased with the concentration of xanthan gum and κ-carrageenan. Thus, the stability and physicochemical properties of protein-polysaccharides are mainly related to the ratios of protein to polysaccharides, salts, pH, temperature and molecular weight of polysaccharide [6,8]. While lots of papers mainly focused on complex coacervates with oppositely charged protein and polysaccharides, a few focused on the same charged ones.

Cyclodextrin (CD) is a torus-shaped oligosaccharide obtained from the degradation of starch by the enzyme cyclodextrin glucosyltransferase [10]. It can be classified based on the number of α-(1,4) bonds linked to glucose units [11]. The most common CDs are the natural α, β and γ-forms, which contain six, seven and eight glucose units, respectively [10]. CDs have a unique chemical appearance, the truncated-cone conformation, which enables it to entrap a variety of guest molecules into its internal hydrophobic cavity to form an inclusion complex. Cui et al. [12] found that CD could mitigate beany odor and reduce the beany flavor of bean-based foods. In addition, CD’s exterior is relatively hydrophilic, while its interior is hydrophobic due to the shielding effect of C–H, making it a good candidate for encapsulating natural bioactive compounds [10]. The functional properties of CD are related to its own structure. The applications of αCD are limited because of its small pore space, which can only contain small guest substances. γCD has large molecular holes, but a high production cost makes it impossible to produce in large quantities in industry. βCD is the most widely used in industry because of its moderate molecular holes, wide application range and low production cost. For example, Pan et al. [13] found that βCD could decrease fish odor of fish gelatin and its gel strength. While, to our knowledge, there is still limited information about how the three kinds of CD interact with FG.

Therefore, in this work, α, β and γ-CD were compared to elucidate the characteristics of the FG–CD complex systems through investigating the gel properties, flow behaviors, ζ-potential, particle size and turbidity. The underlying mechanism was also evaluated by the UV absorption spectrum, far-UV circular dichroism spectroscopy, fluorescence spectroscopy and Fourier transform infrared spectroscopy.

## 2. Results and Discussion

### 2.1. Gel Strength

Gel strength is one of the physical properties of colloids and is considered to be a rigid factor for predicting its physical characteristics [14]. As shown in Table 1, at ratios of 90:10 and 80:20, FG–CD gels have higher gel strength than those of pure gelatin gels (as FG was 4.5% and 4.0%). This might be because that the formation of an FG–CD complex could stabilize the colloids, or the introduction of CDs with lots of –OH could easily interact with proteins to form hydrogen bonds, therefore, enhancing the gels network. Interestingly, with the further increase in CDs (60:40–20:80), FG–CD gels have similar gel strength values to pure gelatin gels (3.0–1.0%). This might be attributed to the introduction of more and more negatively charged CDs, increasing the repulsion force among FG and CDs and weakening gel network (Huang et al., 2018, Sow et al., 2019). Furthermore, according to the reports of Huang et al. (2019) and You et al. (2020), there are three types of intermolecular junctions: FG–FG junctions, FG–CD and CD–CD junctions in an FG–CD mixture. The strengths of the three types junctions are: FG–FG junctions > FG–CD junctions > CD–CD junctions. At low CD concentrations, the gel networks are dominated by FG–FG junctions, along with some reinforcement of FG–CD. At higher CD contents, CD–CD junctions are dominated and gels have some reinforcement from FG–FG junctions. Compared with FG–FG junctions, CD–CD junctions couldn’t form gel. Thus, the proper ratios of FG–CD (90:10 and 80:20) could give FG–CD hydrogels unique gel properties. Moreover, at the same ratios (90:10, 80:20), FG–γCD had the highest gel strength compared with the others; FG–βCD cannot form gels at low ratios of FG–CD (60:40–20:80). This might be because of the different structural and soluble properties of different kinds of CD.

### 2.2. Turbidity, Particle Size, and Zeta Potential

**Figure 1:**
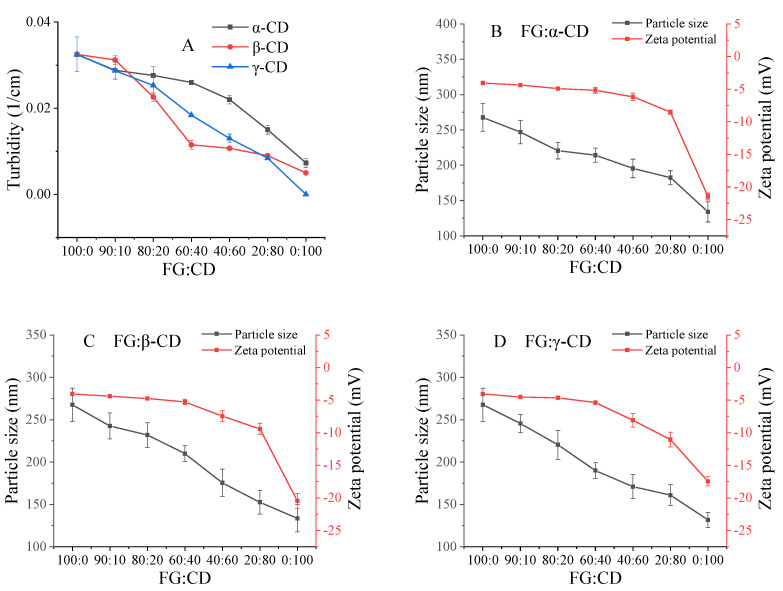
Turbidity, zeta potential and particle size of various FG–CD mixture samples.

The change of turbidity can be used as a sign to evaluate the stability of protein-polysaccharide mixture solutions [3]. Figure 1A shows that a pure FG solution has the maximum turbidity, and the turbidity value presents a downward trend with the increase in CDs. In this paper, the isoelectric point (PI) value of FG was around 5.40. The pH of pure FG, αCD, βCD and γCD solutions were around 5.68, 5.92, 5.90 and 6.00, respectively. Thus, with the increase in CD, the pH of the FG–CD system would be far away from the PI of FG, decreasing turbidity values. Though the turbidity of the pure FG system (0.5–0.1%, *w*/*v*) also showed a decreasing tendency with the decreasing FG concentration, FG–CD systems (FG–CD was 90:10–40:60) still showed slightly higher turbidity values than those of pure FG samples (Data not shown). This demonstrated that FG could interact with CD to form a stable mixture. Combined with the Figure 1B–D, FG has a weak negative charge of −4.05 ± 0.02 mV, while αCD, βCD and γCD have strong negative charges of −21.42 ± 0.48, −20.50 ± 1.20, −17.47 ± 0.68 mV, respectively. Theoretically, both negatively charged FG and CDs should be repulsed by repulsion forces to prevent the formation of complexes [15]. However, particle size shows that the complexation still occurred. Furthermore, the turbidity of the mixtures were all lower than pure FG and there was no stratification in the aqueous solutions, suggesting that the complexes were soluble rather than insoluble. Similarly, Liu et al. [15] found that both negatively charged bovine serum albumin (BSA) and FG could form soluble complexes under certain conditions because there were positive patches on the BSA interacting with carboxyl groups on the FG molecules. In addition, Razzak et al. [3] thought that positively charged patches on the FG molecules induced both net-negatively charged sodium alginate and FG to form soluble complexes. Therefore, we speculated that the negatively charged CD molecules could interact with the specific positively charged regions, which were called positive patches, on the net negative charge FG molecules. In other words, two negatively charged molecules could form a complex with a negatively charged polymer [16].

As shown in Figure 1B–D, the particle sizes of the FG–CD mixture solution decreased with decreasing ratios of FG–CD, suggesting the formation of soluble complexes [3,8,17]. This could also be because the addition of CDs made the large aggregate particles of the FG–CD complex change into small aggregate particles or promoted the dissociation of FG–CD from dimer to monomer [18]. You et al. [4] thought that the particle size of the protein-polysaccharide complex was mainly attributed to the protein-polysaccharide interactions, protein–protein interactions and polysaccharide–polysaccharide interactions. Therefore, at low contents of CDs, FG–CD and FG–FG molecules were dominant in solutions, but with the increase in CD, CD–CD molecules with smaller particle sizes dominated.

Generally speaking, the larger the absolute ζ-potential value is, the more stable the complex system is [9]. Herein, three FG–CD systems had higher absolute ζ-potential values, indicating that the formation of FG–CD complexes was stable and soluble.

### 2.3. Flow Behaviors

**Figure 2:**
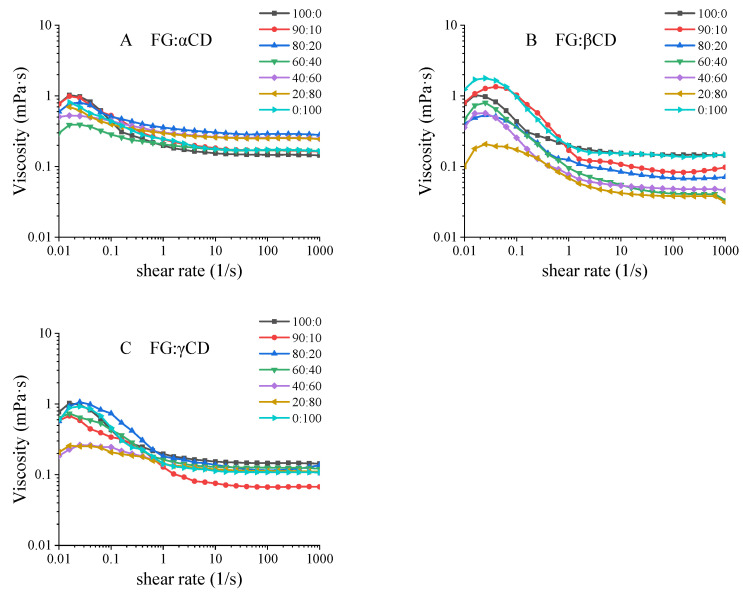
Apparent viscosity of various FG–CD systems.

The viscosity of the gelatin solution is an important technical index of application, which is critical in industrial processing [14]. As shown in Figure 2, the apparent viscosities of all samples increased firstly and then decreased with the increasing shear rate. This indicated that all the aqueous samples exhibited non-Newton behavior at low shear rate and non-Newton behavior at high shear rates. This might be caused by the changes in gel dispersion, molecular shapes or intramolecular chemical bond strength upon shear rates [19]. It is believed that the viscosity at the shear rate of 50 s^−1^ (η_50_) has a good correlation with the sensory thickness, sliminess and stickiness of various foods [9]. As shown in Table S1, FG–αCD mixture solutions have the higher η_50_ than FG, while FG–βCD and FG–γCD mixture solutions have lower η_50_ values, especially for FG–βCD. Combined with the data of turbidity, FG and αCDs are more likely to form large complexes, resulting in stronger resistance to movement.

Furthermore, Power Law, Bingham, Herschel-Bulkley and Cross models were compared to explore the most appropriate model for evaluating the flow behavior of FG–CD mixture solutions [20]. The R^2^ values of all models were >0.99, showing high degrees of correlation. Root Mean Square Error (RMSE) is considered to be the simplest and most informative parameter for linear and nonlinear curve fitting [9]. As shown in Table S1, the Cross model was found to be the most suitable with minimal RMSE values. According to the Power Law and Herschel-Bulkley model, n values of almost all samples were less than 1, showing a pseudo-plastic behavior. Interestingly, this shows relatively great variance compared with Figure 2.

### 2.4. UV Absorption Spectrum Measurements

**Figure 3:**
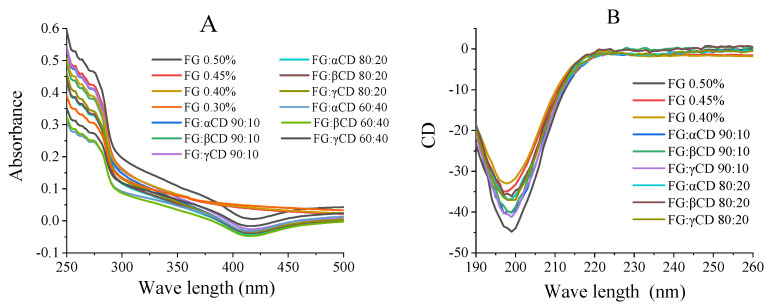
UV absorbance spectra (**A**) and CD spectra of all samples (**B**).

UV spectrum is a practical tool to monitor the relative movement of amino acid residues because some environmentally sensitive amino acids reflect different spectral characteristics [5]. Therefore, the interaction between FG and CD in aqueous state was studied by UV spectroscopy (Figure 3A and Figure S1). FG shows obvious UV absorption near 280 nm, which is related to the vibration of the tyrosine (Tyr) residues [14]. As shown in Figure 3A, the absorbance intensity values of the FG–CD mixtures were lower than their corresponding pure FG samples. Combined with the ζ- potential, both FG and CDs molecules are negatively charged, so the electrostatic repulsion between them may stretch the FG structure, resulting in the decrease in intensity values. Moreover, the formed complex might also decrease the emitted intensity of the UV vis spectrum. Figure S1 also shows that the UV values of FG–CD mixture solutions decreased with the increase in CD contents. This may also be attributed to the dissolution and inclusion of FG into the nonpolar cavity of CDs by the hydrophobic interaction with the increase in CD concentration [11].

### 2.5. Far-UV Circular Dichroism Measurements

Circular dichroism is the most widely used method for the determination of protein secondary structure. As shown in Figure 3b, pure FG solution has one negative band in the far-UV region peaked at 198 nm, which relates to random content [4]. The mean residue ellipticity (MRE) of three different systems decreased gradually with the increase in CDs (Figure 3B and Figure S2), indicating that the structure of FG was changed. Compared with pure FG samples, the addition of CDs increased the negative ellipticity of the FG–CD mixture. In addition, the secondary structure of the samples was analyzed by self-contained software to obtain the proportions of α-helix, β-sheet, β-turn and random coil. As shown in Table 2, for pure FG samples, the higher the contents of FG, the higher β-turn and lower random coil contents. At the same FG (0.45–0.30%) contents, the addition of CDs significantly increased β-turn content but decreased random coil, demonstrating the conformation transition from random coil to β-turn. Moreover, FG–βCD groups have the highest β-turn contents and FG–γCD groups have the highest β-sheet contents. The higher the β-sheet content and the lower the random coil content, the more ordered and stable the protein structure. Thus, at the same concentration of FG (0.45%, 0.40%), FG–αCD, FG–βCD and FG–γCD hydrogels have the higher gel strength than those of pure FG (0.45%, 0.40%) (Table 1).

### 2.6. Fluorescence Spectroscopy

**Figure 4:**
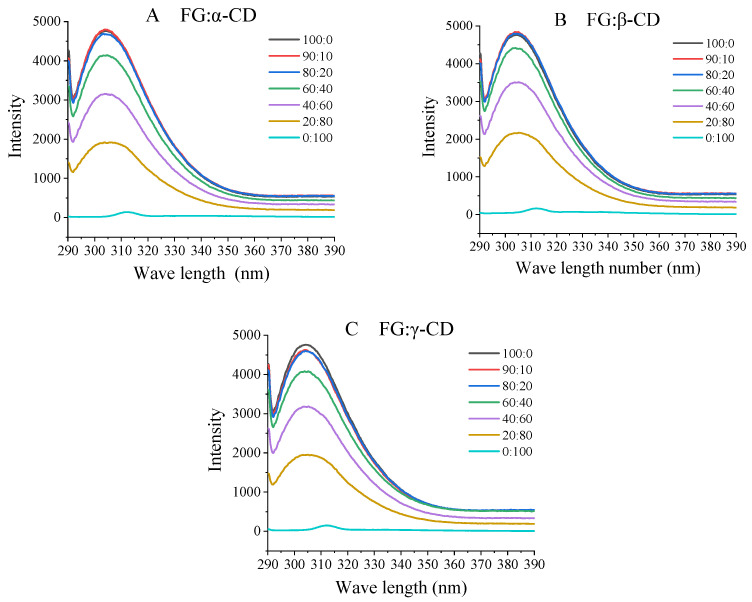
Fluorescence spectra of various FG–CD samples.

Fluorescence spectroscopy could be used to evaluate changes in the protein tertiary conformation [5,18]. The intrinsic fluorescence behavior of gelatin could be attributed mostly to Tyr, with Phe being stable and not excited in most cases [4]. As shown in Figure 4 and Table S2, compared with the pure FG groups, the addition of CDs could increase the intensity of FG. This might be because the introduction of CDs unfold the FG molecule, exposing lots of tryptophan, tyrosine and phenylalanine. For the three FG–CD groups, the tendency of fluorescence intensity firstly increased (FG–CD = 90:10) with the increase in CD contents and then decreased. This might be because of the decrease in fluorophores with the decreasing FG concentration. Furthermore, the amino acid of FG might enter into the CD’s cavity, which then results in the fluorescence quenching. Interestingly, it is worth noting that no matter what the ratio of FG to CD is, the enhancement of the FG–βCD mixture’s fluorescence value is always the most obvious. This is probably due to the structure of βCDs making FG easily expose fluorescence groups to the hydrophobic environment. Nevertheless, the maximum emission wavelength remained constant. This was attributed to the segregative interactions rather than the associative interactions, resulting in an increase in the local concentration of FG, which meant Tyr was exposed to a more hydrophobic environment to enhance the fluorescence intensities [4].

### 2.7. Fourier Transform Infrared (FTIR) Spectroscopy

**Figure 5:**
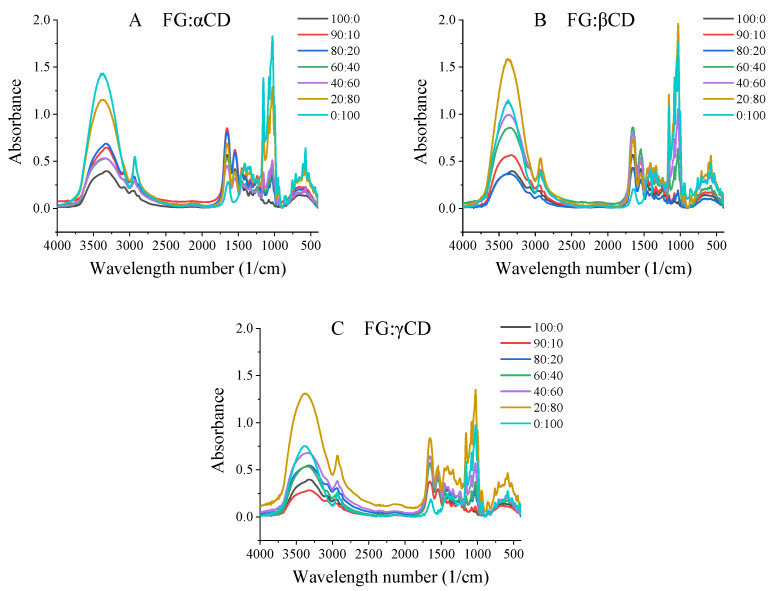
FTIR spectra of various FG–CD samples.

FTIR is a useful technique for characterizing the secondary structure of proteins and investigating molecular interactions [8]. Protein molecules show many vibrational frequencies, which are reflected in different absorption bands in the infrared spectra of proteins [21]. As shown in Figure 5 and Table S3, the spectrum of FG exhibits characteristic absorption bands at 1656 cm^−1^, 1549 cm^−1^, 1240 cm^−1^, 3327 cm^−1^ and 2958 cm^−1^, which are related to Amide I, Amide II Amide III, Amide A and Amide B, respectively. Pure αCD exhibited main IR peaks at 1028, 1078, 1155, 1334, 1640, 2930 and 3367 cm^−1^, associated with C–O–C, C–C, C–O, –OH, C–O, CH– from CH2 and –OH stretching vibrations, respectively [12,22,23]. βCD and γCD were slightly different from αCD, but their peak intensities had significant differences (α > β > γ), decreasing with the increase in molecular weight, which was also consistent with the study of [22].

Amide Ι is the most sensitive spectral region, which is derived from the C=O stretching vibrations of the peptide bond coupled with the in-phase bending of the N–H bond and stretching of the C–N bond [21]. For the FG–CD complex, the wavelength numbers of Amide I were about 1656–1658 cm^−1^, which was slightly higher than that of pure FG (1655.59 cm^−1^), while at the ratio of 20:80, FG–βCD had the lowest wavenumber, which was suspected to be affected by the βCD contents. Amide II is mainly attributed to the in-plane N–H bending and the C–N stretching vibrations, and it could reflect the change in α-helical structure [13,21]. It should be noted that CDs have no characteristic peak here (Table S3), so the wavelength changes of mixtures can well reflect the effect of CDs on the structure of FG. Compared with pure FG, both the FG–βCD group and FG–γCD group had lower Amide II wavelength numbers, while the FG–αCD groups had higher values. These indicated that the addition of βCDs and γCDs changed the secondary structure of gelatin through increasing α-helix contents with the reduction in β-turn (Table S4). Amide III is derived from the C–N stretching vibrations, N–H bending vibrations and the wagging vibration of CH2 in glycine backbone and proline side-chains [13]. For the three FG–CD groups at high CDs contents (FG–CD ranged from 40:60 to 0:100), slightly higher Amide III wavenumbers were observed. This might prove that intermolecular hydrogen bonds are formed between FG and CDs molecules [24].

Amide A is due to the hydrogen bonding stretching vibrations including N–H and O–H [12,17]. In terms of FG–αCD groups, Amide A shifted to a higher wave number (3326–3348 cm^−1^) towards the O–H stretch peak (3367 cm^−1^). This indicated that the intermolecular hydrogen bonds were formed between the O–H groups in αCDs and the N–H groups of FG [8]. Similar results can be obtained from the other two FG–CD groups. Amide B corresponds to the asymmetric and symmetric CH2 stretching vibrations [25]. In the three FG–CD groups, the Amide B declined from 2958 to 2930, 2924 and 2932 cm^−1^ with the increase in αCD, βCD and γCD, respectively. These shifts may be due to the van der Waals interaction between FG and CDs [13].

In addition, the Amide I band (1600–1700 cm^−1^) could be divided into four main components, as follows: 1600–1640, 1640–1650, 1650–1660 and 1660–1700 cm^−1^, which represents β-sheet, unordered, α-helix and β-turn, respectively [12]. To further analyze these differences, Amide I was deconvoluted to analyze secondary structures by PF.EXE software, and the component peaks were well fitted (R2 > 0.999). As shown in Table S4, the three FG–CD groups have higher α-helix contents than that of pure FG, and at the same ratios of FG–CD, FG–γCD groups had the highest β-sheet contents, which were also similar with the CD results (Table S1). These might explain why FG–γCD groups had a higher gel strength than that of FG at the same FG concentration. Moreover, at ratios of 90:10 and 80:20, both FG–βCD and FG–γCD had higher α-helix contents than those of FG–αCD. The higher the α-helix content, the higher the gel strength (Table 1).

## 3. Conclusions

This work comparatively evaluated the interaction among the same negatively charged FG and three different kinds of CD (αCD, βCD and γCD) in an aqueous solution. It showed that proper CDs could improve the gel strength of FG–CD hydrogels, especially for γCD. FG could interact with CDs to form stable soluble complexes with lower values of turbidity, particle size and ζ-potential. Rheological results showed that the αCD could increase the viscosity of FG-αCD mixture solution, while βCD and γCD decreased viscosity, and the Herschel-Bulkley model might be the best for analyzing the flow behaviors of FG–CD groups. Fluorescence spectroscopy results indicated that CDs could make the Tyr expose to a more hydrophobic environment to enhance the fluorescence intensities. CD results showed that CDs mainly transformed the conformational structure of FG from a random coil to β-turn. FTIR results revealed that CDs interacted with the gelatin’s molecules to form a hydrogen bond, which could attribute hydrogels with stable and higher α helix and β-sheet contents.

## 4. Methods

### 4.1. Materials

Fish gelatin (FG, type B, 89.21% protein) was obtained from Shanghai Yuanye Bio-Technology Co., Ltd. (Shanghai, China). α-Cyclodextrin (αCD, purity ≥ 98.0%, MW 972.84) was purchased from Shanghai Macklin Biochemical Co., Ltd. (Shanghai, China). β-Cyclodextrin (βCD, purity ≥ 98.0%, MW 1134.98) was purchased from Beijing Solarbio Science & Technology Co., Ltd. (Beijing, China). γ-Cyclodextrin (γCD, purity ≥ 98.0%, MW 1297.12) was purchased from Shanghai Yuanye Bio-Technology Co., Ltd. (Shanghai, China). Potassium bromide (KBr) was obtained by Beijing J & K Scientific Ltd. (Beijing, China).

### 4.2. Sample Preparation

For gel preparation, FG powder was dissolved in the distilled water in a water bath at 50 °C for 1 h and then cooled to 35 °C, followed by the addition of a certain amount of CD until total biopolymer concentration reached 5% (*w*/*w*). Considering the solubility of αCD, βCD and γCD was 50, 18.5 and 232 mg/mL respectively, the samples were finally divided into different groups as follows: FG–αCD group and FG–γCD group: the ratios of FG to CD were 100:0, 90:10, 80:20, 60:40, 40:60 and 20:80, while FG–βCD groups were FG to CD 100:0, 90:10 and 80:20. The total biopolymer concentrations of FG were 5%, 4.5%, 4%, 3%, 2%, 1%, respectively and the pure FG gels (5%, 4.5%, 4%, 3%, 2%, 1%) were regarded as control. The mixture solutions containing CDs were placed at 25 °C until they were completely dissolved with 100 rpm stirring and 15 mL solution was poured into 25 mL glass beaker, and incubated at 10 °C for 16–17 h.

For aqueous solution preparation, under the condition of 0.5% total concentration, FG–αCD, FG–βCD and FG–γCD groups were prepared at the ratios of FG–CD under 100:0, 90:10, 80:20, 60:40, 40:60, 20:80 and 0:100.

### 4.3. Gel Strength

Gel strength of the above prepared FG–CD groups and gelatin gel samples were measured using a profile texture analyzer (Stable Micro System, Surrey, UK). The samples were measured rapidly with pre-test/testing/post-test speeds of 1 mm/s and gel penetration distance of 4 mm by a *p*/0.5 R probe [9].

### 4.4. Turbidity

Absorbance values of FG–CD aqueous solution were measured at 600 nm using a UV-Vis spectrophotometer (TU-1810, Persee, Beijing, China), and absorbance was transformed into turbidity (τ, cm^−1^) using following Equation (1)

(1) $$\tau =-\left(\frac{1}{L}\right)\ln\left(\frac{I}{I_{0}}\right)$$

where *L* is the light path length of 1 cm, *I* is the transmitted radiation intensity, and *I*_0_ is the intensity of incident radiation. The deionized water was used as reference [8].

### 4.5. Zeta Potential and Particle Size Measurements

The characteristics of ζ-potential and particle sizes of FG–CD solution were performed using a particle microelectrophoresis instrument (Zetasizer Nano ZS90, Malvern Instruments, UK) [26]. The samples were diluted 50-fold with deionized water, and each sample was measured at room temperature.

### 4.6. Flow Behaviors

Flow behaviors of FG–CD aqueous solution were measured using a controlled stress rheometer (AR2000ex, TA Instrument Company, New Castle, DE, USA) with a parallel-plate geometry (60 mm flat plate) according to the methods of Huang et al. [20] with slight modifications. All samples were performed over a range of shear rate (0.01–1000 s^−1^) at 25 °C with a gap of 1 mm. The Power law, Bingham, Herschel-Bulkley and Cross models were comparably used to calculate the flow behavior of the FG-CD mixture solution.

(2) $$\mathrm{Power}\;\mathrm{law}:\;\tau ={\kappa \gamma }^{n}$$

(3) $$\mathrm{Binghan}:\;\tau =\tau_{0}+\eta_{0}\gamma$$

(4) $$\mathrm{Herschel}–\mathrm{Bulkley}:\;\tau =\tau_{0}+\kappa \gamma^{n}$$

(5) $$\mathrm{Cross}:\;\eta =\eta_{\infty }+\frac{\eta_{0}-\eta_{\infty }}{1+{\left(\lambda \gamma \right)}^{m}}$$

where *τ* is the shear stress (mPa), *κ* is the consistency coefficient (mPa·s), *γ* is the shear rate (1/s), *n* means the flow behavior index, *τ*_0_ is the yield stress (mPa), *η*_0_ is the zero-shear viscosity (mPa·s), *m* is the dimensionless exponent, *η_∞_* is the infinitive shear viscosity (mPa·s), *λ* is the time constants related to the relaxation times of the polymer in solution.

### 4.7. UV Absorption Spectrum Measurements

A UV-Vis spectrophotometer (TU-1810, PERSEE, Beijing, China) was used to measure the UV absorption spectrum of FG–CD mixture solutions. The samples were performed from 500 to 250 nm using quartz cells with a 1.0 cm optical path at room temperature. The scan speed was 200 nm/min. The deionized water was used as reference [26].

### 4.8. Far-UV Circular Dichroism Measurements

Far-UV circular dichroism measurements of samples were performed using a Circular dichroism spectrometer (J-1500, JASCO Corporation, Tsukuba, Japan), which was equipped with a 0.1 cm path length quartz cell [4]. The circular dichroisms of all the samples were carried out from 190 to 260 nm with a bandwidth of 1 nm. The samples were diluted 20 times with deionized water before measurements and the distilled water was used for blank control.

### 4.9. Fluorescence Spectroscopy

Fluorescence experiments were carried out by a spectrophotometer (F-4700, HITACHI, Japan) equipped with a quartz cuvette of 1 cm light path length. The excitation wavelength was 280 nm, while the emission wavelength was recorded from 290 to 490 nm. The slit widths of both excitation and emission were set at 5 nm, and the voltage was 360 V with the scan speed set at 12,000 nm/min [5].

### 4.10. Fourier Transform Infrared (FTIR) Spectroscopy

The lyophilized samples were mixed with KBr, ground and pressed into a pellet. A Fourier transform infrared spectrometer (FT/IR-4700, JASCO Corporation, Tsukuba, Japan) was used to collect the FTIR spectra. All spectra were recorded at the range of 4000–400 cm^−1^ and an average of 16 scans at a resolution of 4 cm^−1^. The background was obtained against pure KBr and baseline was corrected with the aid of the software [8].

### 4.11. Statistics Analysis

One-way analysis of variance (ANOVA) and Duncan’s multiple range test were carried out to analyze the differences between samples using SPSS 25.0 (SPSS Inc., Chicago, IL, USA). Significant differences among samples were taken as *p* < 0.05. All measurements were performed in triplicate at room temperature and were expressed as mean values ± standard deviation (SD).

## Figures and Tables

**Table 1:** Gel strength (g) of different FG–CD systems and contents of FG.

Pure FG (*w*/*v*)	5%	4.50%	4%	3%	2%	1%
	299.63 ± 13.31 ^Af^	229.02 ± 12.31 ^Ae^	154.16 ± 1.82 ^Ad^	100.63 ± 2.81 ^Ac^	51.6 ± 1.24 ^Bb^	3.04 ± 0.00 ^Aa^
FG–CD	100:00:00	90:10:00	80:20:00	60:40:00	40:60	0.888889
FG–αCD	299.63 ± 13.31 ^Af^	267.14 ± 11.01 ^Be^	184.43 ± 23.00 ^Bd^	104.49 ± 12.51 ^ABc^	46.05 ± 3.80 ^Ab^	7.44 ± 0.88 ^Ca^
FG–βCD	299.63 ± 13.31 ^Abc^	283.93 ± 12.83 ^BCb^	194.29 ± 10.77 ^Ba^	-	-	-
FG–γCD	299.63 ± 13.31 ^Af^	292.81 ± 10.18 ^CDe^	223.44 ± 10.46 ^Cd^	101.84 ± 14.32 ^ABc^	49.21 ± 4.30 ^ABb^	5.03 ± 1.35 ^Ba^

The values are mean ± SD, calculated from triplicate measurements. Values with different letters in the same column (A–D) and same line (a–f) are significantly different at *p* < 0.05. Pure FG: pure FG gels with 5%, 4.5%, 4%, 3%, 2%, 1% (*w*/*v*) gelatin. As total content of biopolymer is 5% (*w*/*v*), the solubility of βCD is 18.5 g/L (25 °C). βCD could not be dissolved in FG–βCD solution as FG–βCD was 60:40–0:100.

**Table 2:** Secondary structure of various FG–CD systems and FG solution by CD spectra.

Content	Various Ratios	β-Sheet (%)	Random Coil (%)	α-Helix (%)	β-Turn (%)
FG	0.50%	27.37 ± 0.21 ^Bbc^	65.60 ± 0.44 ^Aab^	0 ± 0 ^Aa^	7.07 ± 0.29 ^D^
	0.45%	26.30 ± 0.10 ^Ab^	69.73 ± 0.06 ^Bc^	0 ± 0 ^Aa^	3.97 ± 0.12 ^BCa^
	0.40%	26.37 ± 0.25 ^A^	69.87 ± 0.25 ^BC^	0 ± 0 ^Aa^	3.77 ± 0.55 ^Ba^
	0.30%	27.73 ± 0.06 ^Cb^	71.10 ± 0.52 ^Dd^	0 ± 0 ^Aa^	1.20 ± 0.52 ^Aa^
FG–αCD	90:10:00	27.20 ± 0.10 ^Ab^	65.77 ± 0.29 ^Ab^	0 ± 0 ^Aa^	7.00 ± 0.36 ^BCbc^
	80:20:00	27.30 ± 0.10 ^ABc^	66.10 ± 0.27 ^ABab^	0 ± 0 ^Aa^	6.60 ± 0.17 ^ABbc^
	60:40:00	27.83 ± 0.21 ^Cbc^	65.80 ± 0.25 ^Ab^	0 ± 0 ^Aa^	6.37 ± 0.12 ^Ac^
FG–βCD	90:10:00	26.17 ± 0.35 ^Ba^	65.77 ± 0.60 ^ABabc^	0 ± 0 ^Aa^	9.17 ± 0.51 ^BCd^
	80:20:00	25.17 ± 0.40 ^Aa^	66.57 ± 0.35 ^ABb^	0 ± 0 ^Aa^	8.07 ± 0.83 ^Ad^
	60:40:00	26.47 ± 0.31 ^BCa^	65.13 ± 0.35 ^Aa^	0 ± 0 ^Aa^	8.43 ± 0.64 ^ABd^
FG–γCD	90:10:00	28.13 ± 0.13 ^ABd^	65.10 ± 0.10 ^Aa^	0 ± 0 ^Aa^	6.80 ± 0.17 ^BCb^
	80:20:00	28.07 ± 0.08 ^Ad^	65.60 ± 0.40 ^ABa^	0 ± 0 ^Aa^	6.33 ± 0.31 ^Bb^
	60:40:00	28.03 ± 0.12 ^Ac^	66.17 ± 0.29 ^BCbc^	0 ± 0 ^Aa^	5.80 ± 0.17 ^Ab^

The values are mean ± SD, calculated from triplicate measurements. Values with different letters in the same system. (A–D) and same contents of FG (a–d) are significantly different at *p* < 0.05.

## Data Availability

The data presented in this study are available in Supplementary Materials.

## Supplementary Materials

The following are available online at https://www.mdpi.com/article/10.3390/gels7040260/s1. Figure S1: UV absorbance spectra of various FG–CD systems; Figure S2: CD spectra of various FG–CD systems; Table S1. Flow behavior of various FG–CD systems; Table S2: Fluorescence peak values of various FG–CD systems and FG contents; Table S3: FTIR Amide bands of various FG–CD systems; Table S4: Secondary structure analysis of various FG–20 CD systems by FTIR.

## References

[1] Patino J.M.R., Pilosof A.M. (2011). Protein-polysaccharide interactions at fluid interfaces. Food Hydrocoll..

[2] Souza CJ F., Garcia-Rojas E.E. (2016). Interpolymeric complexing between egg white proteins and xanthan gum: Effect of salt and protein/polysaccharide ratio. Food Hydrocoll..

[3] Razzak M.A., Kim M., Chung D. (2016). Elucidation of aqueous interactions between fish gelatin and sodium alginate. Carbohydr. Polym..

[4] You G., Niu G., Long H., Zhang C., Liu X. (2020). Elucidation of interactions between gelatin aggregates and hsian-tsao gum in aqueous solutions. Food Chem..

[5] Zou W., Mourad F.K., Zhang X., Ahn D.U., Cai Z., Jin Y. (2020). Phase separation behavior and characterization of ovalbumin and propylene glycol alginate complex coacervates. Food Hydrocoll..

[6] Lu X., Xie S., Wang L., Xie H., Fang W. (2020). Electrostatic-driven structural transformation in the complexation of lysozyme and κ-carrageenan. Chem. Phys..

[7] Razavi M.S., Golmohammadi A., Nematollahzadeh A., Fiori F., Farris S. (2020). Preparation of cinnamon essential oil emulsion by bacterial cellulose nanocrystals and fish gelatin. Food Hydrocoll..

[8] Sow L.C., Toh NZ Y., Wong C.W., Yang H. (2019). Combination of sodium alginate with tilapia fish gelatin for improved texture properties and nanostructure modification. Food Hydrocoll..

[9] Huang T., Tu Z., Shangguan X., Wang H., Zhang L., Bansal N. (2020). Characteristics of fish gelatin-anionic polysaccharide complexes and their applications in yoghurt: Rheology and tribology. Food Chem..

[10] Matencio A., Navarro-Orcajada S., García-Carmona F., López-Nicolás J.M. (2020). Applications of cyclodextrins in food science. A review. Trends Food Sci. Technol..

[11] Sivakumar K., Parinamachivayam G., Murali Krishnan M., Chakravarty S., Bharathi A. (2018). Preparation, characterization and molecular modeling studies of the beta-cyclodextrin inclusion complex with benzoguanamine and its analytical application as chemosensor for the selective sensing of Ce^4+^. Spectrochim. Acta Part A Mol. Biomol. Spectrosc..

[12] Cui L., Kimmel J., Zhou L., Rao J., Chen B. (2020). Combining solid dispersion-based spray drying with cyclodextrin to improve the functionality and mitigate the beany odor of pea protein isolate. Carbohydr. Polym..

[13] Pan J.F., Hui J., Shang M., Qi L., Chang X., Yao W., Hao W., Dong X. (2018). Effects of deodorization by powdered activated carbon, β-cyclodextrin and yeast on odor and functional properties of tiger puffer (*Takifugu rubripes*) skin gelatin. Int. J. Biol. Macromol..

[14] Huang T., Zhao H., Fang Y., Lu J., Yang W. (2019). Comparison of gelling properties and flow behaviors of microbial transglutaminase (MTGase) and pectin modified fish gelatin. J. Texture Stud..

[15] Liu J., Shim Y.Y., Wang Y., Reaney MJ T. (2015). Intermolecular interaction and complex coacervation between bovine serum albumin and gum from whole flaxseed (*Linum usitatissimum* L.). Food Hydrocoll..

[16] Park J.M., Muhoberac B.B., Dubin P.L., Xia J. (1992). Effects of protein charge heterogeneity in protein-polyelectrolyte complexation. Macromolecules.

[17] Zhan F., Ding S., Xie W., Zhu X., Chen Y. (2020). Towards understanding the interaction of β-lactoglobulin with capsaicin: Multi-spectroscopic, thermodynamic, molecular docking and molecular dynamics simulation approaches. Food Hydrocoll..

[18] Huang T., Tu Z., Wang H., Shangguan X., Zhang L., Zhang N.-H., Bansal N. (2017). Pectin and enzyme complex modified fish scales gelatin: Rheological behavior, gel properties and nanostructure. Carbohydr. Polym..

[19] Huang T., Tu Z., Zou Z., Shangguan X., Bansal N. (2020). Glycosylated fish gelatin emulsion: Rheological, tribological properties and its application as model coffee creamers. Food Hydrocoll..

[20] Yang H., Yang S., Kong J., Dong A., Yu S. (2015). Obtaining information about protein secondary structures in aqueous solution using Fourier transform IR spectroscopy. Nat. Protoc..

[21] Chen L.F., Shen Q., Shen J.P., Shi D.T., Chen T., Yu H.R. (2012). Studies and comparison of the liquid adsorption and surface properties of α-, β- and γ-cyclodextrins by FTIR and capillary rise method. Colloids Surf. A Physicochem. Eng. Asp..

[22] Gu Z.J., Zhang Q.C., Shen Q. (2015). Synthesis and comparison of polyaniline nanofibers templated by α-, β- and γ-cyclodextrin. J. Polym. Res..

[23] Fan L., Du Y., Huang R., Wang Q., Wang X., Zhang L. (2005). Preparation and characterization of alginate/gelatin blend fibers. J. Appl. Polym. Sci..

[24] Huang S., Tu Z.C., Sha X.M., Wang H., Hu Z.Z. (2020). Gelling properties and interaction analysis of fish gelatin–low-methoxyl pectin system with different concentrations of Ca^2+^. LWT-Food Sci. Technol..

[25] Bonenfant D., Niquette P., Mimeault M., Furtos-Matei A., Hausler R. (2009). UV-VIS and FTIR spectroscopic analyses of inclusion complexes of nonylphenol and nonylphenol ethoxylate with β-cyclodextrin. Water Res..

[26] Huang T., Tu Z., Shangguan X., Wang H., Sha X. (2018). Gelation kinetics and characterization of enzymatically enhanced fish scale gelatin–pectin coacervate. J. Sci. Food Agric..

