# Supplementary material for: Interaction between Negatively Charged Fish Gelatin and Cyclodextrin in Aqueous Solution: Characteristics and Formation Mechanism
Source: Gels. 2021 Dec 13;7(4):260. doi: 10.3390/gels7040260 (PMC8701615; doi:10.3390/gels7040260)
Supplement: Supplementary file 1 [file gels-07-00260-s001.zip › gels-1451674-supplementary.pdf]

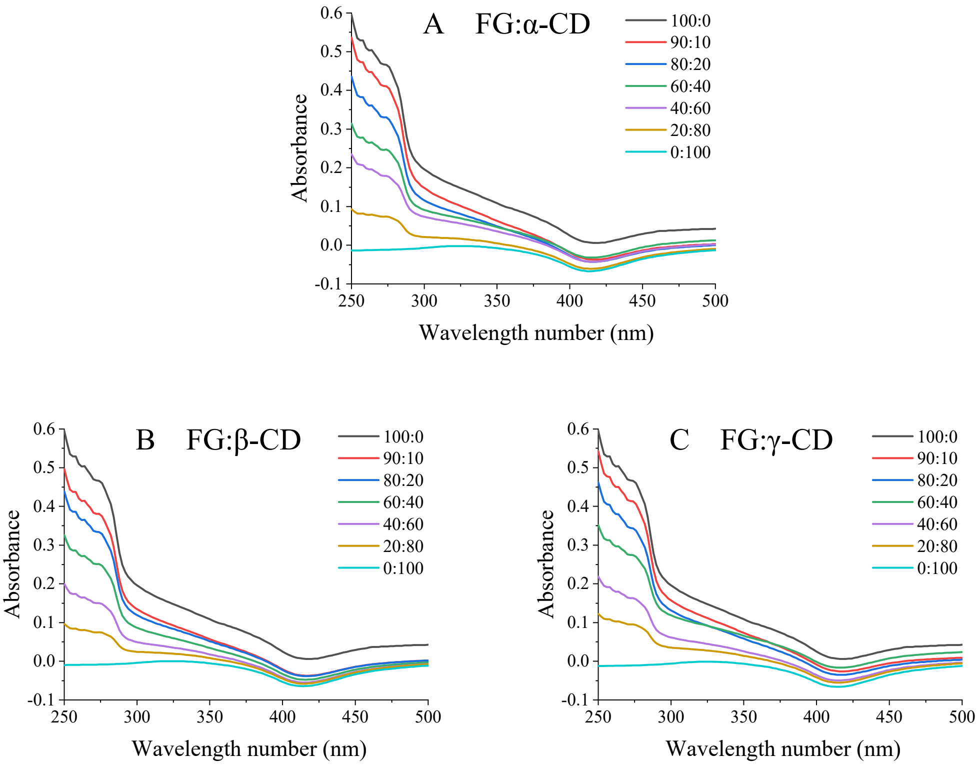

Figure S1. UV absorbance spectra of various FG-CD systems

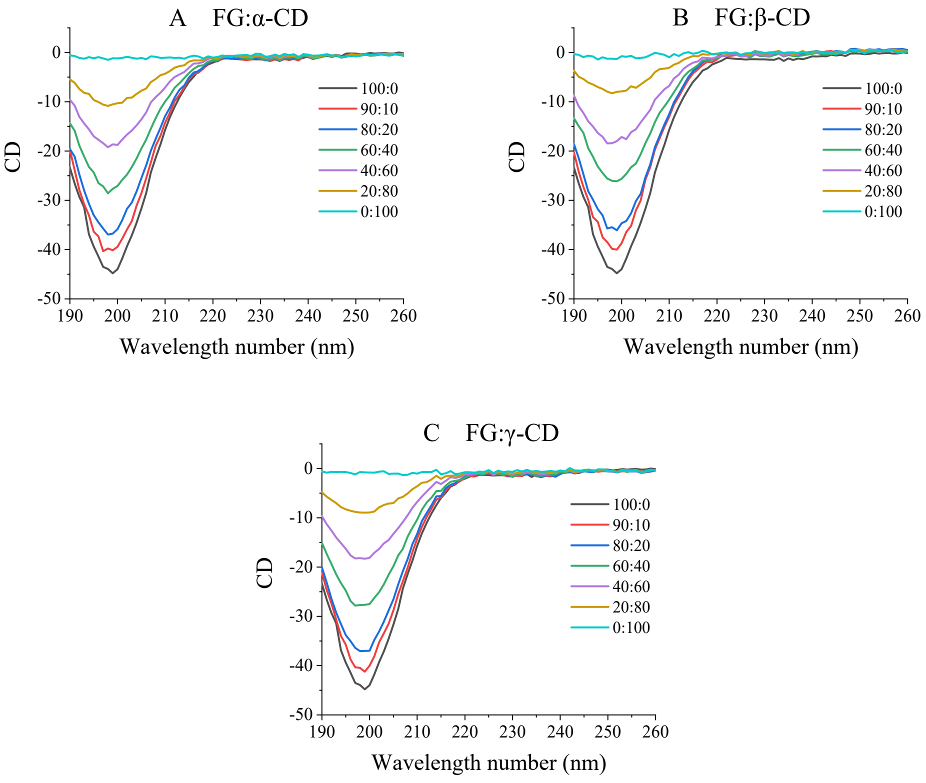

Figure S2. CD spectra of various FG-CD systems

Table S1. Flow behavior of various FG-CD systems

	Various ratios	η_{50} (mPa· s)	Power low			Binghan			Herschel-Bulkley				Cross		
			RMSE	κ (mPa· s)	n	RMSE	τ_0 (mPa)	η_0 (mPa· s)	RMSE	τ_0 (mPa)	κ (mPa· s)	n	RMSE	η_0 (mPa· s)	m
FG: αCD	100:0	0.15±0.01	0.152	0.16	0.98	0.296	0.127	0.144	0.151	-0.015	0.16	0.98	0.039	0.056	1.013
	90:10	0.17±0.00	0.150	0.19	0.98	0.456	0.232	0.164	0.150	-0.010	0.19	0.98	0.039	0.061	1.001
	80:20	0.28±0.01	0.442	0.33	0.98	0.871	0.305	0.282	0.431	-0.120	0.33	0.98	0.085	0.086	1.018
	60:40	0.17±0.07	0.234	0.19	0.98	0.455	0.154	0.166	0.228	-0.067	0.19	0.98	0.038	0.043	1.017
	40:60	0.26±0.00	0.370	0.29	0.98	0.786	0.284	0.249	0.359	-0.110	0.30	0.97	0.065	0.063	1.015
	20:80	0.25±0.07	0.407	0.29	0.98	0.763	0.245	0.246	0.395	-0.122	0.29	0.98	0.070	0.068	1.021
	0:100	0.17±0.03	0.254	0.20	0.97	0.596	0.232	0.167	0.246	-0.074	0.20	0.97	0.042	0.045	1.011
FG: βCD	90:10	0.12±0.05	0.384	0.05	0.99	1.088	-0.282	0.095	0.310	0.273	0.046	0.99	0.320	0.279	1.112
	80:20	0.07±0.04	0.197	0.04	0.98	0.316	-0.019	0.070	0.167	0.013	0.04	0.99	0.177	0.134	1.041
	60:40	0.03±0.01	0.608	0.09	0.86	0.903	0.292	0.036	0.601	-0.119	0.10	0.85	0.306	0.155	1.247
	40:60	0.05±0.01	0.136	0.06	0.97	0.204	0.079	0.047	0.136	-0.007	0.06	0.97	0.077	0.049	1.039
	20:80	0.04±0.01	0.589	0.08	0.86	0.848	0.224	0.034	0.578	-0.140	0.09	0.85	0.278	0.111	1.272
	0:100	0.03±0.01	0.327	0.11	0.97	0.739	-0.165	0.146	0.279	0.209	0.11	0.97	0.300	0.225	1.051
FG: γCD	90:10	0.10±0.02	0.074	0.07	0.99	0.070	0.037	0.068	0.069	0.032	0.07	0.99	0.041	0.059	1.023
	80:20	0.12±0.01	0.498	0.07	0.98	1.550	-0.479	0.133	0.426	0.326	0.07	0.98	0.441	0.320	1.114
	60:40	0.13±0.01	0.168	0.14	0.98	0.372	0.150	0.122	0.165	-0.037	0.14	0.98	0.032	0.042	1.013
	40:60	0.11±0.01	0.248	0.13	0.98	0.393	0.136	0.109	0.246	-0.036	0.13	0.97	0.126	0.068	1.030
	20:80	0.11±0.01	0.035	0.12	0.99	0.161	0.094	0.111	0.035	0.006	0.12	0.99	0.035	0.024	0.997
	0:100	0.11±0.03	0.064	0.11	0.99	0.094	0.056	0.108	0.063	0.017	0.11	0.99	0.027	0.044	1.009

Table S2. Fluorescence peak values of various FG-CD systems and FG contents

	100:0	90:10	80:20	60:40	40:60	20:80
FG	$4770.00±36.17^{Af}$	$4527.67±13.65^{Ae}$	$4355.33±11.59^{Ad}$	$4017.00±22.52^{Ac}$	$3093.67±16.65^{Ab}$	$1908.33±3.51^{Aa}$
FG:αCD	$4770.00±36.17^{Ae}$	$4811.33±24.17^{Cef}$	$4694.67±35.02^{Cd}$	$4147.67±18.61^{Cc}$	$3162.67±10.02^{Bb}$	$1927.67±23.25^{ABa}$
FG:βCD	$4770.00±36.17^{Ae}$	$4853.67±8.74^{Df}$	$4808.00±32.79^{Dd}$	$4421.67±22.59^{Dc}$	$3523.00±14.00^{Db}$	$2178.67±11.59^{Ca}$
FG:γCD	$4770.00±36.17^{Af}$	$4642.00±12.12^{Be}$	$4606.33±7.23^{Bd}$	$4085.33±10.11^{Bc}$	$3199.00±12.12^{Cb}$	$1960.33±10.50^{Ba}$

The values are mean ± SD, calculated from triplicate measurements. Values with different letters in the same column (A-D) and same line (a-f) are significantly different at $p < 0.05$

Table S3. FTIR Amide bands of various FG-CD systems

	Various ratios	Amide I	Amide II	Amide III	Amide A	Amide B
FG: αCD	100:0	1655.59	1548.56	1240.00	3326.61	2958.27
	90:10	1655.59	1548.59	1240.00	3328.71	2938.98
	80:20	1655.59	1549.52	1240.00	3330.61	2938.02
	60:40	1656.55	1550.49	1241.93	3332.39	2935.13
	40:60	1658.48	1550.49	1241.93	3345.89	2933.20
	20:80	1656.55	1551.45	1242.90	3347.82	2931.27
	0:100	1640.16	-	1241.93	3367.10	2930.31
FG: βCD	90:10	1657.52	1541.81	1240.00	3336.25	2954.41
	80:20	1657.52	1541.81	1240.97	3355.53	2936.09
	60:40	1657.52	1542.77	1240.97	3342.03	2935.13
	40:60	1658.48	1542.77	1242.90	3359.39	2929.34
	20:80	1649.80	1543.74	1243.86	3376.75	2927.41
	0:100	1650.77	-	1245.79	3359.39	2923.56
FG: γCD	90:10	1656.55	1543.74	1240.00	3327.57	2941.88
	80:20	1657.52	1542.77	1240.97	3328.53	2938.02
	60:40	1657.52	1541.81	1240.97	3339.14	2936.09
	40:60	1658.48	1541.81	1241.93	3338.18	2934.16
	20:80	1657.52	1542.77	1242.90	3376.75	2932.23
	0:100	1657.52	-	1241.93	3389.28	2932.23

Table S4. Secondary structure analysis of various FG-CD systems by FTIR

Various ratios		β-sheet	Random coil	α-helix	β-turn
	100:0	31.96±0.31^{bc}	18.28±0.56^{ab}	9.65±0.12^{a}	40.11±0.30^{a}
FG: αCD	90:10	31.34±0.14^{a}	18.48±0.23^{ab}	9.92±0.12^{bc}	40.26±0.26^{ab}
	80:20	31.76±0.13^{b}	18.21±0.01^{a}	9.80±0.07^{ab}	40.24±0.08^{a}
	100:0	31.96±0.31^{bc}	18.28±0.56^{ab}	9.65±0.12^{a}	40.11±0.30^{c}
FG: βCD	90:10	31.69±1.22^{b}	18.01±0.47^{a}	12.81±0.45^{c}	37.49±0.14^{a}
	80:20	30.12±1.25^{a}	19.52±0.91^{bc}	12.23±0.05^{b}	38.14±0.40^{b}
	100:0	31.96±0.31^{a}	18.28±0.56^{b}	9.65±0.12^{a}	40.11±0.30^{c}
FG: γCD	90:10	36.95±0.05^{c}	12.13±0.13^{a}	12.31±0.06^{c}	38.62±0.12^{b}
	80:20	33.96±0.17^{b}	19.14±0.06^{c}	10.47±0.13^{b}	36.44±0.37^{a}

The values are mean ± SD, calculated from triplicate measurements. Values with different letters in the same system (a-c) are significantly different at $p < 0.05$.
